# Hyaluronic acid-based nano-sized drug carrier-containing Gellan gum microspheres as potential multifunctional embolic agent

**DOI:** 10.1038/s41598-018-19191-7

**Published:** 2018-01-15

**Authors:** Ming Fang Hsu, Yen Sheng Tyan, Yu Chen Chien, Ming Wei Lee

**Affiliations:** 1 Department of Medical Laboratory and Biotechnology, Chung Shan Medical University, Taichung, Taiwan; 2 Department of Radiology, Chung Shan Medical University Hospital, Taichung, Taiwan; 3 Department of Clinical Laboratory, Chung Shan Medical University Hospital, Taichung, Taiwan

## Abstract

The purpose of this study was to develop a gellan gum-based multifunctional embolic agent. Calibrated spherical gellan gum and nanoparticle-containing gellan gum microspheres were prepared via water-in oil emulsification method. Self-assembled nanoparticles composed of short-chain hyaluronic acid and polyethylenimine as the doxorubicin carrier were prepared. The short-chain hyaluronic acid/polyethylenimine/ doxorubicin (sHH/PH/Dox) with the mean size was 140 ± 8 nm. To examine sHH/PH/Dox nanoparticle uptake into cells, the results confirmed that sHH/PH nanoparticles as drug carrier can facilitate the transport of doxorubicin into HepG2 liver cancer cells. Subsequently, sHH/PH/Dox merged into the gellan gum (GG) microspheres forming GG/sHH/PH/Dox microsphere. After a drug release experiment lasting 45 days, the amount of released doxorubicin from 285, 388, and 481 μm GG/sHH/PH/Dox microspheres were approximately 4.8, 1.8 and 1.1-fold above the IC50 value of the HepG2 cell. GG/sHH/PH/Dox microspheres were performed in rabbit ear embolization model and ischemic necrosis on ear was visible due to the vascular after 8 days. Regarding the application of this device in the future, we aim to provide better embolization agents for transcatheter arterial chemoembolization (TACE).

## Introduction

Transarterial embolization (TAE) involves the introduction of an embolic agent, either spherical or non-spherical, into the blood vessel to achieve localized occlusion of blood flow^1^. This type of treatment typically applies to liver cancer, prostatomegaly, arteriovenous malformation, preoperative tumor embolism, acute hemorrhage, gastrointestinal bleeding, or tumor-induced hemorrhage^2^. TAE is an invasive technique widely applied in the clinical setting, especially when, for example, liver metastasis occurs resulting in a tumor size of >3 cm, at which point the patient can neither undergo surgical procedure nor adjuvant chemotherapy (equivalent to Stage B in Barcelona Clinic Liver Cancer staging system)^3^. Taking the role of transarterial embolization (TAE) in the treatment of hepatocellular carcinoma (HCC) as an example, the underlying principle of TAE is described as follows^4^. The liver has a unique dual blood supply from both the portal vein and the hepatic artery. The normal parenchyma of the liver receives two-thirds of its necessary blood supply from the portal vein and receives the remaining one-third from the hepatic artery. However, it is well-known that vascularization of hepatocellular carcinoma (HCC) is mostly (90–95%) dependent on the hepatic artery. Embolic agents are injected into the hepatic artery which blocks blood flow, blood supply to liver cancer cell is cut off and induce cell necrosis. However, the blood supply to the normal liver tissue is still maintained by dominant blood flow from the portal vein minimizing damage to the liver. The difference between transarterial embolization (TAE) and transarterial chemoembolization (TACE) is the noninvolvement or involvement of chemotherapy drugs with embolic agents^5^. Unlike systemic chemotherapy through intravenous injection (drugs given through the whole body), in TACE, chemotherapeutic drugs are released from the embolic agent and allow a higher dose to the tumor tissues. TACE can prevent systemic side effects caused by drug-induced damage to other normal cells in the body. Regarding treatment for enlarged prostate, prostatic artery embolization (PAE) is a relatively novel approach involving the use of microcatheter to inject embolic agents via small prostatic arteries into the prostate gland, which blocks the prostatic artery and subsequently obstructs the blood supply to the enlarged prostate, causing tissue necrosis. Thus, the enlarged prostate shrinks, reducing the volume of the prostate gland and decrease the risk of urethral strictures^6^.

According to a market survey announced by AB Newswire in December 2016, the embolization-related agents and devices market is expected to reach US$450 million by 2024^7^. There are two types of embolic agents: mechanical occlusion device and flow-directed embolic agents^8^. Mechanical occlusion device includes metal coils and detachable balloon, which are used in hemorrhagic or aneurysmal conditions through deployment in the target vessel to reduce blood flow and platelet aggregation, thereby achieving the effect of embolization. Flow-directed embolic agents include particulates (microsphere), polymers, or *in-situ* gelling^9^. This type of agent is transferred using a catheter to the peripheral vessels of the target tissue, blocking the blood supply to the target tissue (cancer tissues) and causing atrophy of the target tissue. Majority of commercial embolic agents are non-degradable such as Onyx®, Embosphere®, DC Bead® and others^10^. Non-degradable embolic agents are used in the embolization of liver cancer, which often causes liver tissue infarction at non-tumor sites or causes gallbladder infarction. In addition, non-degradable embolic agents often use artificial polyvinyl alcohol and its derivatives as substrate^11^, which easily causes adverse reactions in patients. Therefore, this study aimed to develop a multifunctional transarterial chemoembolization agent using degradable carbohydrate (gellan gum, GG) as the matrix. The target application for this embolization agent was TACE for liver cancer.

In our previous studies, we verified the characteristics of gellan gum and make a suitable starting point for implantable biomedical devices^12,13^. In the present experiment, we adopted gellan gum as the substrate for developing a drug-carrier microspherical embolization agent. Gellan gum was prepared into microspheres by using emulsification. We also synthesized short-chain hyaluronic acid-histidine (sHH) and polyethylenimine-histidine (PH) copolymers, both of which can self-assemble into nanoparticle and application as drug carrier^14^. The nano drug carriers were combined with doxorubicin (Dox) according to the principle of electrical property, forming sHH/PH/Dox nanoparticles. Doxorubicin is a chemotherapeutic drug currently used to treat liver cancer^15^. Subsequently, sHH/PH/Dox nanoparticles merged into the gellan gum microspheres, forming multifunctional GG/sHH/PH/Dox microsphere. The characteristics, drug release kinetic and vascular embolization efficiency of the embolic agent were examined.

## Results and Discussion

### Preparation of gellan gum (GG) microspheres

**Figure 1:**
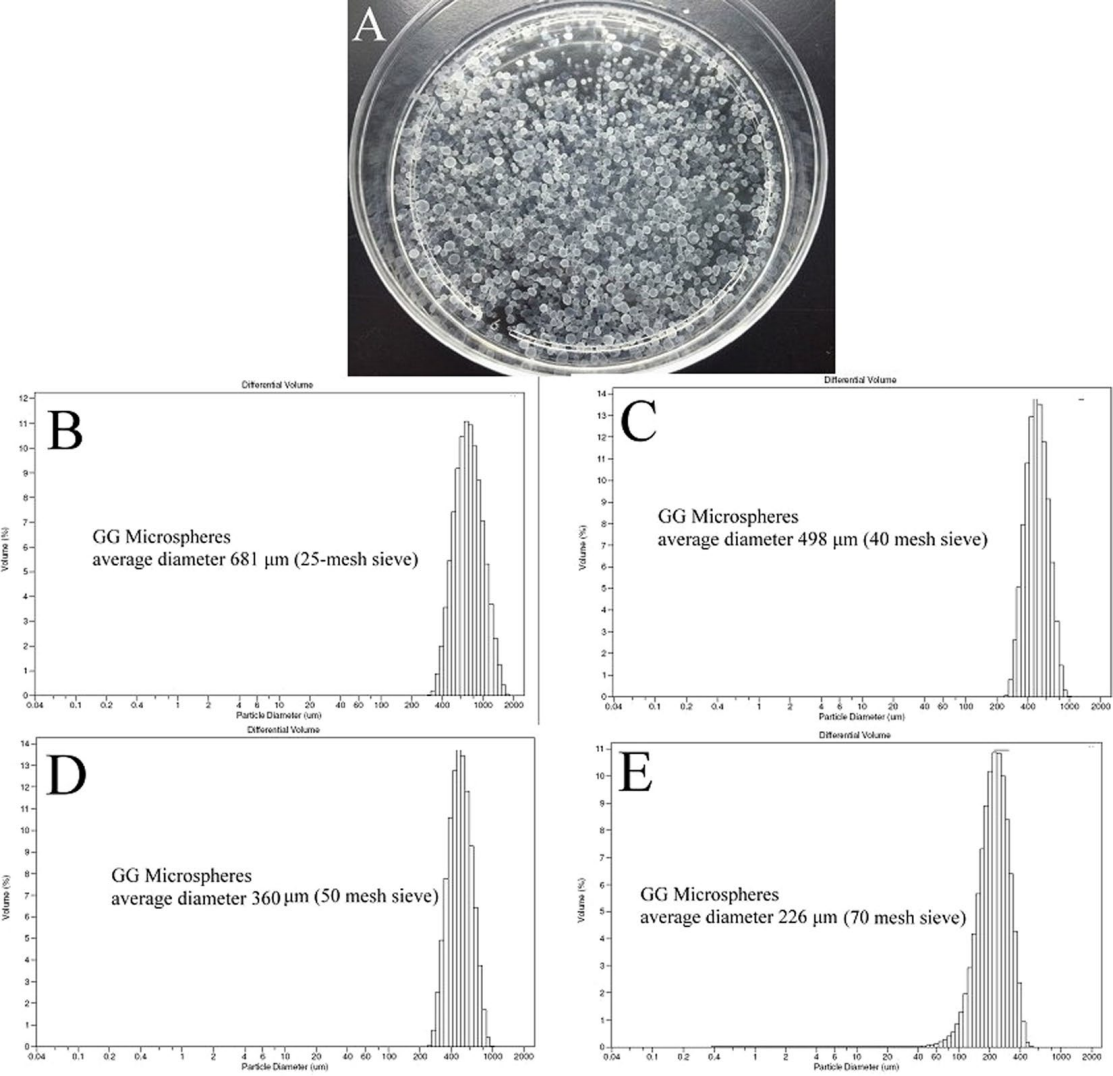
Morphology of the GG microspheres (**A**). GG microspheres size distribution of using 25-mesh sieve (**B**), 40-mesh sieve (**C**), 50-mesh sieve (**D**) and 70-mesh sieve (**E**).

The size of embolic microspheres depends on the size of the blood vessel to be embolized. In clinical practices, embolic microspheres generally range between 50 nm and 900 nm in diameter, whereas the size of microspheres used for embolization of liver cancer ranges from 50 μm to 500 μm in diameter^16^. The most suitable microspheres for use in active embolization are based on hydrophilic Polymeric microspheres are typically prepared by the emulsion method. The size of emulsion-based microspheres is influenced by many parameters, including polymer concentration, rotation speed, type of surfactant used, temperature of emulsification, and viscosity of the oil phase. For the purpose of preparing gellan gum (GG) microspheres of varying sizes, the concentration of gellan gum was used as a parameter. Different concentrations of gellan gum (0.5%, 0.4%, 0.3% w/v) were reacted in 50 °C mineral oil (400 ml) at 400 rpm for 10 min. Subsequently, 1.25% (w/v) calcium chloride solution (10 ml) and Span85 (1 ml) were added to the mixture and mixed for 1 h. Morphology of the GG microspheres were shown in Fig. 1A.

Thereafter, the mixture was sieved in 25, 40, 50 and 70-mesh sieves, respectively, to separate the GG microspheres with different size. According to results of the particle analyzer, a 25-mesh sieve can first separate GG microspheres over 500 μm (681 ± 51 μm, Fig. 1B) from the mixture, and 40, 50 and 70-mesh sieves can obtain GG microspheres of 498 ± 32 (Fig. 1C), 360 ± 24 (Fig. 1D) and 226 ± 15 (Fig 1E) μm, respectively. Emulsification was performed with different concentrations of gellan gum and the weight percent of the different sizes of GG microspheres by sieving using standard sieves were shown in Table 1.

**Table 1:** The GG microspheres size distribution and weight percent of various GG concentration of emulsion.

	681 μm (25 mesh)	498 μm (40 mesh)	360 μm (50 mesh)	226 μm (70mesh)	Other small size (200~50 μm)
0.5% GG	66%	17%	2%	2%	13%
0.4% GG	51%	16%	8%	5%	20%
0.3% GG	35%	15%	9%	11%	30%

The results indicated that when gellan gum in different concentrations was emulsified, different amount of microspheres of varying sizes was obtained. High gellan gum concentration resulted in considerable amount of large microspheres. This result can be explained using the Ostwald Ripening theory^17^. When polymer concentration is high, smaller crystals in the solute or sol particles dissolve and redeposit onto larger particles, causing an increase in the amount of larger particles. The goal of this study was to develop an embolization agent for liver cancer therapy; therefore, we aimed at obtaining microspheres of <500 μm. When 0.5% gellan gum was emulsified, 34% of the gellan gum microspheres were <500 μm; when 0.4% gellan gum was emulsified, 49% of the gellan gum microspheres were <500 μm; and when 0.3% gellan gum was emulsified, 65% of the gellan gum microspheres were <500 μm. Their results suggested that used the 0.3% gellan gum for emulsification allowed to get the higher percentage of <500 μm microspheres. We also tried the lower gellan gum solution on emulsion and proof that when the gellan gum concentration was less than 0.3%, it was difficult to form GG microspheres.

**Figure 2:**
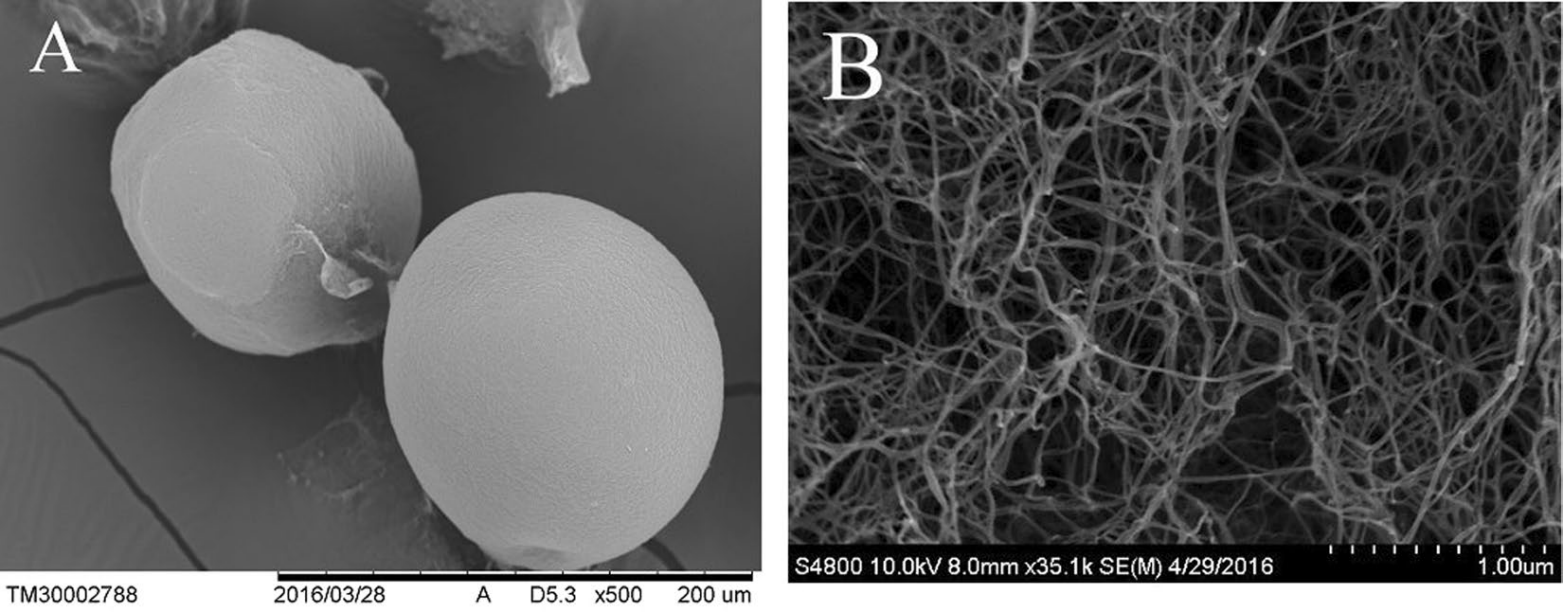
SEM images of surface morphologies of GG microspheres, 500× (**A**) and 3500× (**B**).

The micrographs of the external surface of the GG microspheres are presented in Fig. 2A. At low magnification (500×) the GG microspheres presented spherical shape and had a smooth surface. At high magnification (3500×) the GG microspheres had rough and porous surfaces (Fig. 2B). The results showed that the GG microspheres are composed of gellan gum fibers. Also, GG microspheres with unique porous structure (pore size 1~7 μm), is suitable for drug delivery^18^. Arterial embolic agent may lead to thrombosis. Thrombosis is due to platelet accumulation. The MPV (mean platelet volume) and PDW (platelet distribution width) of platelet are 6.8~13.5 μm^3^ and 9~19 μm^3^. In this study, gellan gum microspheres with porous structure (pore size 1~7 μm), the pore size was small that did not cause platelet accumulation.

### Characterization of short-chain hyaluronic acid-histidine (sHH) polymer

To prepare short-chain hyaluronic acid/polyethylenimine nano-sized drug carriers, short-chain hyaluronic acid and polyethylenimine were covalently bonded to histidine. Histidine contains an imidazole ring, which acts as a proton provider or proton acceptor under different pH conditions. At pH 7.4, histidine as proton provider could react with the acidic group of short-chain hyaluronic acid by covalent bonds, forming short-chain hyaluronic acid-histidine (sHH) copolymer. sHH is the amphiphilic polymer with hydrophilic (short-chain hyaluronic acid) and hydrophobic (histidine) residues. At pH 7.4, histidine as proton acceptor could react with the primary amine of polyethylenimine by covalent bonds, forming polyethylenimine-histidine (PH) copolymer. PH is also an amphiphilic polymer with hydrophilic (histidine) and hydrophobic (polyethylenimine) residues. sHH and PH self-assembled into sHH/PH nanoparticles through hydrophobic/hydrophilic interactions^14,19^.

**Figure 3:**
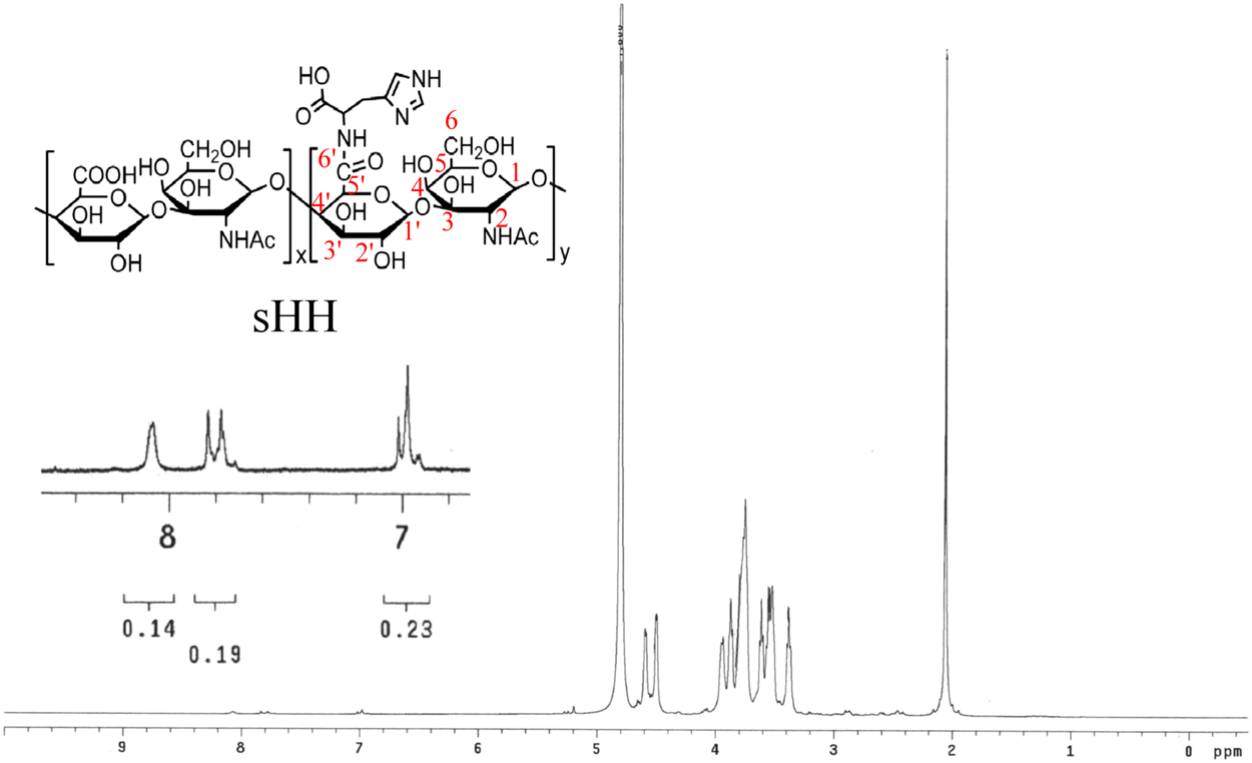
^1^H NMR spectrum of sHH polymer.

The chemical structures of sHH were characterized by ^1^H NMR spectrum (Fig. 3). The ^1^H NMR spectrum of the sHH presented the following signals^20,21^: δ7.9 ppm (methenyl protons located in imidazole group of his, -N = CH-), δ7.0 ppm (methenyl protons located in imidazole group of his, -N-CH = C-), δ 4.8 ppm (H1 of N-acetylglucosamine, GlcNAc), δ 4.7 ppm (H1′ of Glucuronic acid, GluA), δ 3.9 ppm (H2 of GlcNAc), δ 3.7–3.8 ppm (H3, H6 of GlcNAc and H4′, H5′ of GluA), δ 3.5–3.6 ppm (H4, H5 of GlcNAc and H3′ of GluA), and δ 3.4 ppm (H2′ of GluA). From the integration of peak area, the degree of substitution (DS) of his to short-chain hyaluronic acid was calculated to be 10%.

### Characterization of polyethylenimine-histidine (PH) polymer

**Figure 4:**
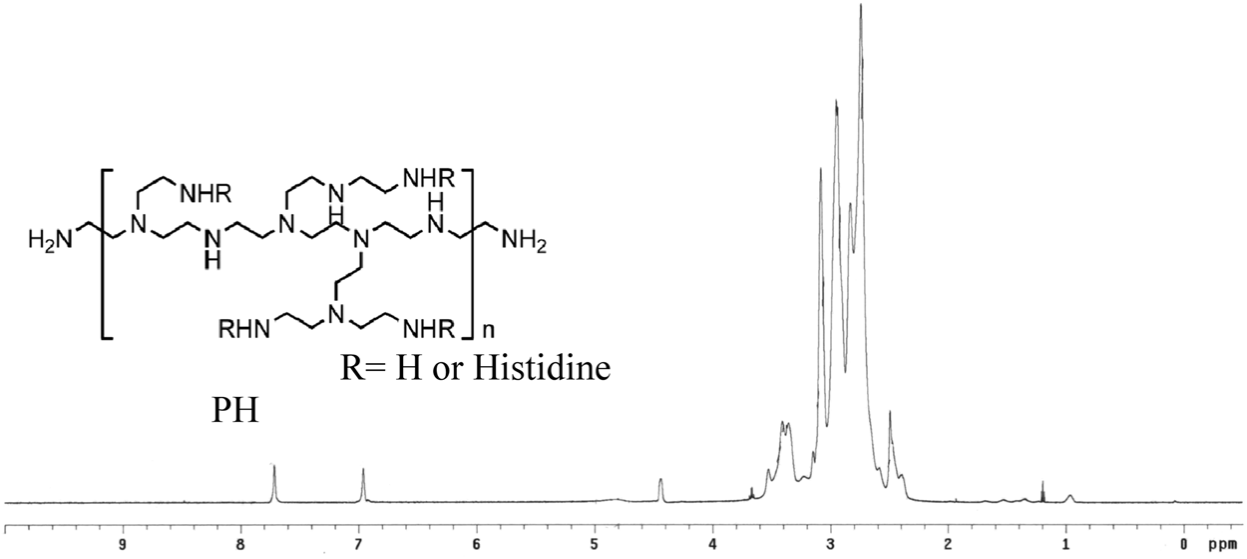
^1^H NMR spectrum of PH polymer.

Polyethylenimine (PEI) was grafted with histidine to yield PH polymer, an amphiphilic molecular. The 1H-NMR spectrum (Fig. 4) of PH has peaks atδ2.4–3.1 ppm due to methylene protons of PEI. The peak at δ7.7 ppm and δ 6.9 ppm are referred to the methenyl protons located in imidazole group of his (-N = CH-, -N-CH = C-). The peak at δ 3.4 ppm was referred to the –CH2- protons of his (Lin *et al*., 2017). As calculated, 20% amino groups in PEI were conjugated with histidyl groups.

### Characterization of sHH/PH/Dox nanoparticles

Doxorubicin (Dox) is the one of the most commonly used drugs for the treatment of both hematological and solid tumors. Doxorubicin is an anticancer drug most often used in treating persistent tumor growth. But its use is limited owing to its side effect profile, such as hypersensitivity reactions, liver and kidney dysfunction, as well as severe cardiotoxicity. To reduce the side effects of doxorubicin, common practices are used drug carriers system, the main advantage is that it can effectively improve stability of drug, improve patients’ drug absorption capacity, and prevent high local drug concentrations, which can effectively extend action time of drug, then effectively improve clinical effect of the drug and minimize toxic and side effects of the drug^22,23^. Dox drug delivery systems that have been developed include polymers, polypeptides, encapsulated particles and nanoparticles^24^. Different carrier-drug interaction mechanisms have been explored, and they can be broadly sub-divided into three types: simple encapsulations, electrostatic interactions and covalent conjugations^25^. In this study, we attempted to make use of sHH/PH nanoparticles as carriers and bound them to doxorubicin through electrostatic interaction. PH was prepared into 1 mg/ml solution, with zeta potential measuring 45.1 mV. sHH was prepared into 1 mg/ml solution, with zeta potential measuring −42.2 mV. When sHH and PH are mixed, sHH and PH self-assemble into sHH/PH nanoparticles because of their hydrophilic/hydrophobic properties. In this study, sHH and PH were mixed at 2:1 ratio, resulting in sHH/PH nanoparticles with surface potential approximating zero. sHH and PH were mixed at 4:1 ratio, forming sHH/PH nanoparticles with surface potential measuring −25.4 mV. Doxorubicin is a type of anthracycline with positively charged, which can therefore bind to the negatively charged sHH/PH nanoparticles^26^. Particle size analysis revealed that the mean size of sHH/PH/Dox was 140 ± 8 nm, and the polydispersity index (PDI) was 0.099. The PDI doesn’t greater than 0.7 indicate that the sHH/PH/Dox nanoparticle has a very narrow size distribution. In addition, the drug encapsulation efficiency (EE) and drug loading level (DL) of DOX-loaded sHH/PH nanoparticles were measured to be 40.2 ± 8.1% and 2.8 ± 0.9%, respectively.

### sHH/PH/Dox nanoparticle cell uptake test

**Figure 5:**
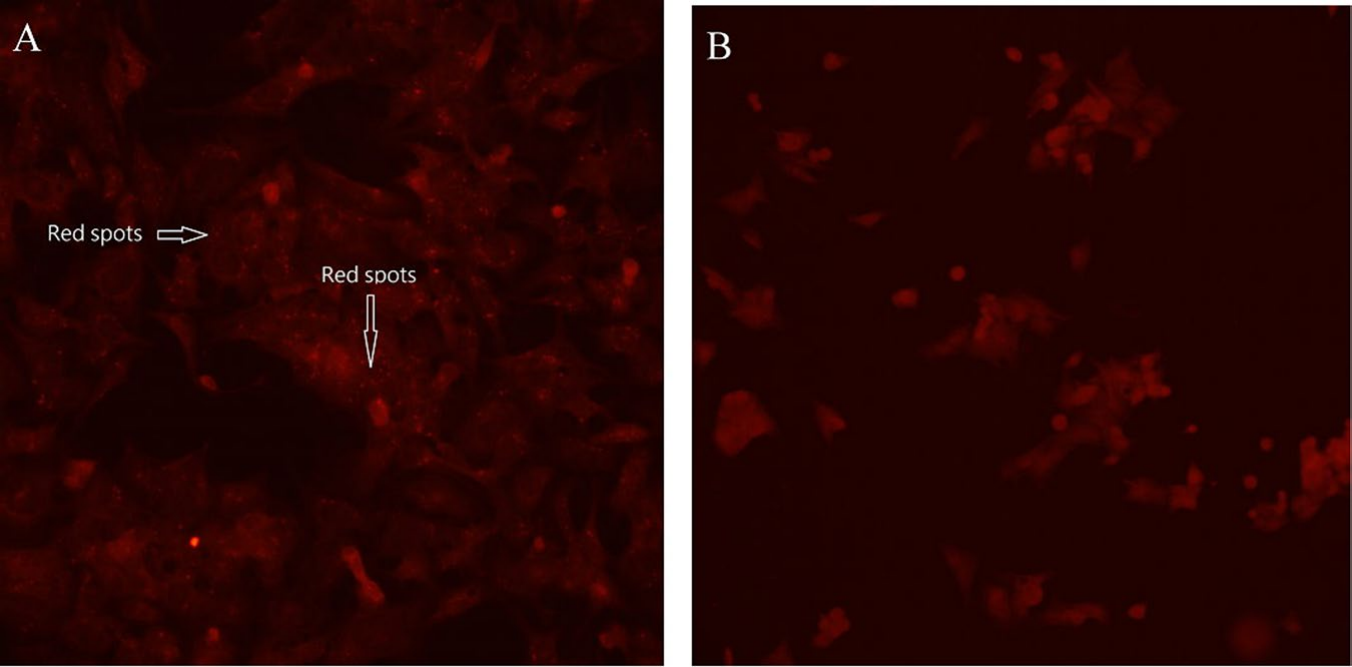
Distribution of sHH/PH/Dox nanoparticles inside HepG2 cells, as shown by inverted fluorescence microscope. sHH/PH/Dox nanoparticles co-culture with HepG2 cells (**A**), Dox alone co-culture with HepG2 (**B**).

The advantage of using sHH/PH nanoparticles as carrier of doxorubicin is that hyaluronic acid can increase its affinity with cancer cells through CD44 receptor^27^. PEI features high transfection capability, which can facilitate the transport of sHH/PH/Dox into cancer cells, increasing drug availability^28^. To verify that sHH/PH/Dox can enter cancer cells, liver cancer cell HepG2 was cultured with SHH/PH/Dox (1 mg/ml) for 24 h. Tumor cells were incubated with the fluorescent SHH/PH/Dox (doxorubicin with fluorescence properties, excitation at 480 nm; emission maximum at 560–590 nm)^29^ and were qualitative using inverted fluorescence microscope. As showed in Fig. 5A, the expected cellular uptake of sHH/PH/Dox nanoparticle (red fluorescence spots) could be found in HepG2 cells. For the control group (Fig. 5B), HepG2 was cultured with doxorubicin alone for 24 h, and the results indicated the absence of doxorubicin in the cell. The results confirmed that the sHH/PH nanoparticles as carrier of doxorubicin can facilitate the transport of doxorubicin into cancer cells.

### Preparation of GG/sHH/PH/Dox microspheres

The results verified that when 0.3% gellan gum was emulsified, the highest yield rate of <500 μm microspheres was observed under this condition. Thus, this experimental condition was selected for the preparation of GG/sHH/PH/Dox microsphere to verify the influence of sHH/PH/Dox nanoparticles on the preparation of gellan gum microspheres. According to results of the particle size analyzer, a 25-mesh sieve can first separate GG/sHH/PH/Dox microsphere >500 μm (674 ± 33) from the mixture, and 40, 50, and 70 mesh sieves can obtain GG/sHH/PH/Dox microspheres of 481 ± 22, 388 ± 32 and 285 ± 17 μm, respectively. To compare the results of gellan gum emulsification with or without sHH/PH/Dox nanoparticles, the sizes distribution of the GG microspheres and GG/sHH/PH/Dox microspheres showed no significant difference (P value < 0.05). The experiment verified that sHH/PH/Dox nanoparticles exist and do not exert considerable influence on the size of gellan gum microspheres. An identical conclusion was reported by Li *et al*.^30^, in which poly (acrylic acid) microspheres were loaded with superparamagnetic iron oxide nanoparticles, and their results verified that the presence of iron oxide nanoparticles does not influence the particle distribution of poly (acrylic acid) microspheres.

### Doxorubicin release studies

**Figure 6:**
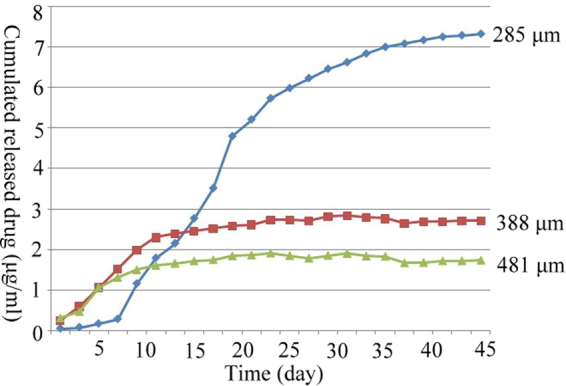
Release profile of Dox from the 285, 388, 481 μm GG/sHH/PH/Dox microspheres in PBS at 37 °C.

The *in vitro* release studies for GG/sHH/PH/Dox microspheres containing Dox were performed using 0 02 M PBS (pH 7.4, 37 °C) as a representative medium^31^. In this study, 285, 388, and 481 μm GG/sHH/PH/Dox microspheres were selected as substrates for doxorubicin drug release. Doxorubicin was quantified using a spectrophotometer at 480 nm. Figure 6 presents the result of doxorubicin release based on different GG/sHH/PH/Dox microsphere sizes. This result indicated that after 45 days of doxorubicin release test, the cumulative amount of release was 7.3 μg/ml, 2.7 μg/ml, and 1.7 μg/ml, suggesting that (1) with the same weight, small GG/sHH/PH/Dox microspheres exhibit a larger surface area, causing accelerated release of doxorubicin; and (2) small GG/sHH/PH/Dox microspheres swell up significantly after absorbing water, which causes an increased rate of doxorubicin release^32^.

In drug release study, the duration of drug release and the amount of drug released are two crucial parameters for clinical application. In TACE, patients generally receive embolization once every one or two month(s). Therefore, a slow rate of degradation in embolization agent is desired, and a long-acting release of the chemotherapeutic drug it carries is anticipated. Because gellan gum degraded slowly, the release of doxorubicin from GG/sHH/PH/Dox microspheres takes 45 days to reach saturation, which satisfies the requirement of TACE. The IC50 of doxorubicin in HepG2 cell is 1.5 μg/ml^33^. The experimental results showed that the amount of released doxorubicin from 285, 388, and 481 μm GG/sHH/PH/Dox microspheres were approximately 4.8, 1.8 and 1.1-fold above the IC50 value. On the basis of the results observed, we assert that three sizes of multifunctional GG/sHH/PH/Dox microspheres prepared in this study meet the requirements of TACE applications.

### Animal study of vessel embolization

**Figure 7:**
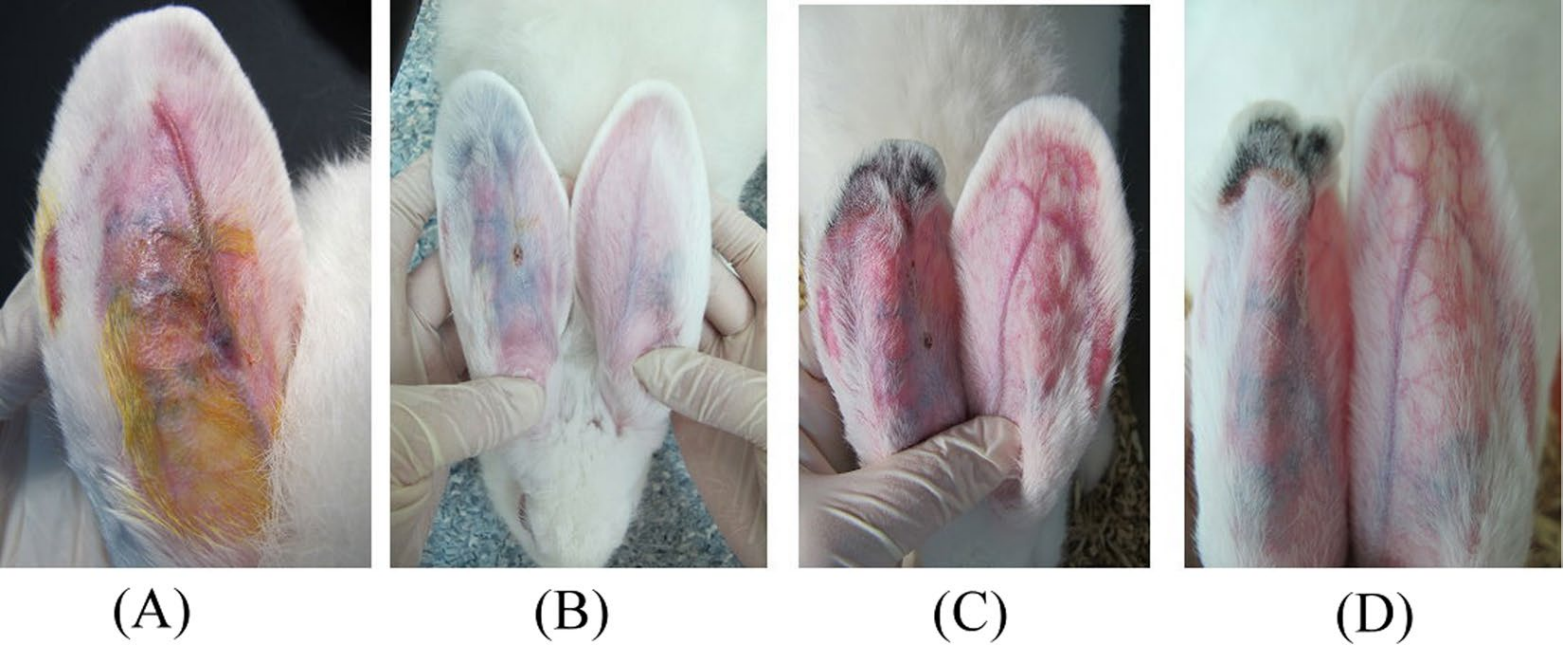
The pre- and post-embolization with GG/sHH/PH/Dox comparison of the rabbits ears in different time (0, 4, 8 and 12 days).

To facilitate assessment of the embolization efficiency of GG/sHH/PH/Dox (285 µm) microspheres *in vivo*, rabbit ear model in our study was used for evaluating the medical embolic agents due to its easy establishment and observed by macrography as well as its low cost and efficacy^34,35^. The results were shown in Fig. 7. The central auricular artery and branch of ear artery was visible before embolization (Fig. 7A, 0 d). After the 4rd day embolization (Fig. 7B), blood flow at the tip of the left ear (experimental group), compared to the right ear (control group) was clogged. In the experimental group, the branches of the artery were no longer visible due to the vascular occlusion caused by GG/sHH/PH/Dox microspheres. In addition, edema and inflammation was not obvious on the experimental group, it was verified that GG/sHH/PH/Dox microspheres with good biocompatibility. After the 8rd day embolization (Fig. 7C), the experimental group, the symptoms of ischemic necrosis with blackened and scabbed on the tip of ear tissue got worst. In the 12th day (Fig. 7D), the arterioles on the tip of ear were atrophy and disappear. The tissue around embolism position was completely ischemic necrosis and because of vascular occlusion caused by the GG/sHH/PH/Dox microspheres. These results of embolization behavior indicated that GG/sHH/PH/Dox microspheres could achieve embolization efficacy, could be used as a potential embolization agent.

In conclusion, various type of embolization agent based on polymer have been developed. For different clinical purposes, the properties of the embolization agent play an important role in guiding clinical choices. In this study, we used gellan gum as the substrate and merged the nano-sized drug delivery system to prepare different sizes GG/sHH/PH/Dox microspheres multifunctional embolic agent. The advantage of using the embolic agent, the gellan gum-based microspheres are suitable for long acting vascular embolization and with excellent biocompatibility. In addition, GG/sHH/PH/Dox microspheres that it can effectively improve stability of doxorubicin, increasing the affinity of drug towards cancer cells and increasing drug availability. Regarding the application of this device in the future, we aim to provide better embolization agents for the treatment of liver cancer or prostatomegaly.

## Methods

### Materials

Gellan gum (G1910), mineral oil (M3516), 1-(3-dimethylaminopropyl)-3-ethylcarbodiimide hydrochloride (EDC, Sigma 1769), EMEM (D5671), sodium pyruvate (P5280) were purchased from Sigma. Span 85 (85549) were purchased from Fluka. N-Hydroxysuccinimide (NHS, 56480), L-Histidine (H8000), Doxorubicin hydrochloride (PHR 1789), Triethylamine (T0886) and Zoletil (Virbac)/Xylazine (46995) were purchased from Sigma-Aldrich. Short-chain Hyaluronic acid (Molecular Weight 12000 Dalton) was purchased from LIFECORE. Branched polyethylenimine (Molecular Weight 25 kDa, 408727) was purchased from Aidrich. Fetal bovine serum (GibcoTM) and gentamycin (Gibco 15710072) were purchased from Gibco. Standard sieves were purchased from Retsch®, Germany. The New Zealand White rabbits obtained from National Laboratory Animal Center (Taipei, Taiwan).

### Preparation of gellan gum (GG) microspheres

Gellan gum microspheres were made by an emulsification and cation-induced cross-linking process^36^. With the aim of controlling microsphere size through manipulation of process and formulation parameters, three different sets of experiments were conducted, where a single factor was varied while keeping the others constant. The aqueous gellan gum (0.3%, 0.4%, 0.5% w/v) solution were prepared by dissolving the gellan gum polymer powder in distilled water by stirring and kept at 85 °C–90 °C until it became a transparent solution, and then the temperature was reduced to 50 °C with further mixing. A 10% (w/w) water-in-oil (W/O) emulsion was then prepared by blending mineral oil (90% w/w) and gellan gum solution (10% w/w) using a high speed (400 rpm) blender for 10 min. Then 1.25% (w/v) CaCl_2_ and 0.5% (w/v) Span 85 were added to emulsification-solution using a syringe with a 23 G hypodermic needle under gentle stirring and then kept at 50 °C, high speed (400 rpm) blender for 60 min. After that, 1.25% (w/v) CaCl_2_ was added again and the gellan gum was allowed to harden for 10 min at 50 °C. Gellan gum microspheres were separated into different size fractions by sieving using 25-mesh (710 µm), 40-mesh (425 µm), 50-mesh (300 µm) and 70-mesh (212 µm) standard sieves. The microspheres retained on each sieve were washed three times with acetone and then collected and weighed. The microsphere size of samples was measured by a laser diffraction particle size analyzer (Mastersizer 2000, Malvern, United Kingdom). Before each measurement, the samples were suspended in pure water and stirred ultrasonically for 10 min to disperse effectively. Every measurement was repeated at least three times. Morphological characterization of the microspheres was carried using scanning electron microscopy (Jeol JSM-6400, Japan).

### Synthesis of short-chain Hyaluronate-Histidine (sHH polymer)

Short-chain Hyaluronic acid 0.66 g was dissolved in 150 ml of distilled water and the pH of the solution was adjusted to 5.5. Then, 1-(3-dimethylaminopropyl) -3-ethylcarbodiimide hydrochloride (EDC) (48.0 mg) and N-Hydroxysuccinimide (NHS) (28.8 mg) were added, the solution was allowed to stir for 30 min. After that, the pH of the mixture was adjusted to 7.4 and L-Histidine (51.8 mg) was then added. The solution was maintained at 25 °C for 24 h. At the end of the reaction, the solution was put into dialysis tube (Spectra/Por membrane MWCO 3500 Da) and subjected to dialysis against distilled water for 3 days to remove the residual EDC, NHS and L-Histidine. The dialyzed short-chain Hyaluronate-Histidine (sHH) solution was freeze-dried, analyzed by ^1^H NMR (500 MHz, Bruker Advance DRX500), and stored as powder.

### Synthesis of Polyethylenimine -Histidine (PH polymer)

L-Histidine (1.32 g) EDC (2.45 g) and NHS (1.47 g) were dissolved in distilled water and the pH was adjusted to 5.5. After 30 min, branched polyethylenimine solution 1 ml was added and the pH of the mixture solution was adjusted to 7.4 and then kept at 25 °C for 24 h. The resulting solution was dialyzed (MWCO 14 kDa) against distilled water for 3 days and the product (PH polymer) was collected by lyophilization. The chemical structure of PH was determined by ^1^H NMR (500 MHz, Bruker Advance DRX500) spectrum.

### Preparation of sHH/PH/Doxorubicin (sHH/PH/Dox) nanoparticles

sHH and PH were dissolved in distilled water, respectively. Then PH solution (1 mg/ml) was added into HH solution (1 mg/ml) dropwise at 2:1 and 4:1 (w/w) ratios to obtain sHH/PH nanoparticles. The mixed solutions were incubated at 25 °C for 30 min to achieve equilibrium. The zeta potential and average size of the nanoparticles were measured by Particle Size and Zeta Potential Analyzer (Malvern, Zetasizer Nano).

Doxorubicin hydrochloride (DOX•HCl, 0.5 mg) and Triethylamine (1 ml) were dissolved in 4 ml of Formamide with stirring in the dark overnight, followed by the addition of sHH/PH nanoparticles (20 mg). The mixture solution was then put into a dialysis tube (MWCO 3500 Da) and subjected to dialysis against PBS (pH 7.4) for 48 h. the PBS was replaced every 8 h to remove organic solvents and unloaded drug. After that, the mixture solution was subjected to dialysis against distilled water for 24 h. DOX-loaded sHH/PH (sHH/HH/Dox) nanoparticles were lyophilization. The zeta potential and average size of the sHH/HH/Dox nanoparticle were measured by Particle Size and Zeta Potential Analyzer (Malvern, Zetasizer Nano). The supernatant obtained after centrifugation was suitably diluted and analyzed for free DOX by UV spectrophotometry (Hitachi U3000, Japan) at 480 nm^37^. The encapsulation efficiency (EE) and drug loading level (DL) of the DOX-loaded sHH/HH nanoparticles were calculated. EE = (mass of loaded drug/mass of feed drug) × 100%, DL = (mass of loaded drug/mass of drug loaded sHH/HH nanoparticles) × 100%.

### HepG2 uptake of sHH/PH/Dox nanoparticle

Human hepatoma cells (HepG2), were used as the model cell line for evaluation of uptake of sHH/PH/Dox nanoparticles. Cells were obtained from the Bioresource Collection and Research Center (BCRC), Taiwan. The cells were grown on EMEM containing 1 mM sodium pyruvate supplemented with 10% fetal bovine serum, and 40 mg/ml gentamycin, at 37 °C. Cells were trypsinized on reaching 90% confluence. Hep G2 cells were seeded on glass coverslips and allowed to adhere by incubating for a period of 24 h at 37 °C. The medium was discarded and replaced with sHH/PH/Dox nanoparticles medium (experimental group) and without sHH/PH/Dox nanoparticles medium (control). The glass coverslips then incubated at 37 °C for 24 h. At the end of 24 h, we washed the glass coverslips with cold PBS three times to remove the excess nanoparticles not taken up by the cells and then fixed the cell with 1% glutaraldehyde. In order to remove residue nanoparticles completely, we used ultrasonic equipment to clean the glass coverslips again. The cells were viewed under the inverted fluorescence microscope (Zeiss Axioskop 2).

### Preparation of GG/sHH/PH/Dox microsphere

The aqueous gellan gum (0.3%, 40 ml) solution with sHH/PH/Dox nanoparticles (0.04 g) was prepared and then the temperature kept at 50 °C with further mixing. A 10% (w/w) water-in-oil (W/O) emulsion was then prepared by blending mineral oil 360 ml and GG/sHH/PH/Dox solution (40 ml) using a high speed (400 rpm) blender for 10 min. Then 1.25% (w/v) CaCl_2_ and 0.5% (w/v) Span 85 were added to emulsification-solution using a syringe with a 23 G hypodermic needle under gentle stirring and then kept at 50 °C, high speed (400 rpm) blender for 60 min. After that, 1.25% (w/v) CaCl_2_ was added again and the GG/sHH/PH/Dox microsphere was allowed to harden for 10 min at 50 °C. GG/sHH/PH/Dox microspheres were separated into different size fractions by sieving using standard sieves (Retsch®, Germany) 25 (710 µm), 40 (425 µm), 50 (300 µm) and 70 (212 µm) meshes. The microspheres retained on each sieve were washed three times with acetone and then collected. The microsphere size of samples was measured by a laser diffraction particle size analyzer. Every measurement was repeated at least three times.

### Drug release studies

The doxorubicin released from different size of GG/sHH/PH/Dox microspheres was performed in 1.5 ml microtest tubes. The GG/sHH/PH/Dox microspheres (1 mg/ml) were placed into the tubes and immersed in 1 ml of phosphate buffer (0.02 M, pH 7.2). Samples (n = 5) were incubated at 37 °C with shaking for 45 days. At defined time points, 1 ml of the release buffer was withdrawn and replaced with fresh buffer. The doxorubicin content was determined by spectrophotometry at 480 nm.

### *In vivo* vessel embolization

Three healthy rabbits (weighing 2.6–3.0 kg) were used for *in vivo* chemoembolization studies. The New Zealand White rabbits obtained from National Laboratory Animal Center (Taipei, Taiwan). The hairs on the marginal ear veins of the rabbits were removed before administration. The New Zealand White rabbits were etherized with Zoletil (Virbac) /Xylazine (20–40 mg/kgZ + 5–10 mg/kgX). Then, the formulations (GG/sHH/PH/Dox microspheres 20 mg suspension in l ml glycerin) were slowly injected into the proximal part of ear veins (0.15 ml/ear), respectively^34^. After administration, the injection site was pressed for 30 s, which could prevent the formulation from spreading through the bloodstream. On 0, 4, 8, 12 days after administration, the macroscopic changes in shape and color of rabbits’ ears were carefully observed to evaluate the chemoembolization effect.

#### Ethics statement concerning animal work

The authors confirm that all experiments were performed in accordance with relevant guidelines and regulations. Animal work methods were carried out in accordance with procedures that were approved by the IACUC (Approval NO 1505: Vaild From 08/01/2016 to 07/31/2018) of the Chung-Shan Medical University Experimental Animal Center.

### Statistical analysis

Each of the experiments was repeated at least three times, and the values were expressed as means ± standard deviations. For comparison between two groups of data, the t-test was performed. Differences were considered to be statistically significant at P < 0.05.

## References

[1] Yu SJ, Yoon JH (2013). Molecular targeted therapy with transarterial chemoembolization. Gastrointest Interv..

[2] Shin SW (2009). The current practice of transarterial chemoembolization for the treatment of hepatocellular carcinoma. Korean J Radiol..

[3] Han K, Kim JH (2015). Transarterial chemoembolization in hepatocellular carcinoma treatment: Barcelona clinic liver cancer staging system. World J Gastroenterol..

[4] Chegai F, Orlacchio A, Merolla S, Monti S, Mannelli L (2015). Intermediate hepatocellular carcinoma: the role of transarterial therapy. Hepat Oncol..

[5] Lanza E (2017). Transarterial Therapies for Hepatocellular Carcinoma. Liver Cancer..

[6] Noor A, Fischman AM (2016). Prostate Artery Embolization as a New Treatment for Benign Prostate Hyperplasia: Contemporary Status in 2016. Curr Urol Rep..

[7] Health & Medicine, http://www.abnewswire.com/pressreleases/transcatheter-embolization-and-occlusion-devices-market-to-exceed-us-45-billion-by-2024_90781.html. (September 10, 2017).

[8] Vaidya S, Tozer KR, Chen J (2008). An overview of embolic agents. Semin Intervent Radiol..

[9] Poursaid A, Jensen MM, Huo E, Ghandehari H (2016). Polymeric materials for embolic and chemoembolic applications. J Control Release..

[10] Wang YXJ, Baere TD, Idée JM, Balley S (2015). Transcatheter embolization therapy in liver cancer: an update of clinical evidences. Chin J Cancer Res..

[11] Liu QS, Mei OL, Li YH (2017). Polyvinyl alcohol terminal chemoembolization for hepatocellular carcinoma with hepatic arteriovenous shunts: Safety, efficacy, and prognostic factors. Eur J Radiol..

[12] Lee MM, Tsai HF, Wen SM, Huang CH (2012). Photocrosslinkable gellan gum film as an anti-adhesion barrier. Carbohydr Polym..

[13] Chang SJ (2012). *In vitro* properties of gellan gum sponge as the dental filling to maintain alveolar space. Carbohydr Polym..

[14] Chen JX, Wang M, Tian HH, Chen JH (2015). Hyaluronic acid and polyethrlenimine self-assembled polyion complexes as pH-sensitive drug carrier for cancer therapy. Colloids Surf B Biointerfaces..

[15] Jayakumar R, Nair A, Rejinold S, Maya S, Nair SV (2012). Doxorubicin-loaded pH-responsive chitin nanogels for drug delivery to cancer cells. Carbohydr Polym..

[16] Porcu EP (2017). Engineered polymeric microspheres obtained by multi-step method as potential systems for transarterial embolization and intraoperative imaging of HCC: Preliminary evaluation. Eur J Pharm Biopharm..

[17] Elbert DL (2011). Liquid-liquid two phase systems for the production of porous hydrogels and hydrogel microspheres for biomedical applications: A tutorial review. Acta Biomater..

[18] Wei Y, Wang Y, Zhang H, Zhou W, Ma G (2016). A novel strategy for the preparation of porous microspheres and its application in peptide drug loading. J Colloid Interface Sci..

[19] Kobayashia Y, Arai N (2016). Self-assembly of Janus nanoparticles with a hydrophobic hemisphere in nanotubes. Soft Matter..

[20] Lee MW, Yang TP, Peng HH, Chen JW (2012). (2012). Preparation and characterization of polygalacturonic acid/rosmarinic acid membrane crosslinked by short chain hyaluronate for preventing postoperative abdominal adhesion. Carbohydr Polym..

[21] Cruz C, Santos SD, Cabrita EJ, Queiroz JA (2013). Binding analysis between l-histidine immobilized and oligonucleotides by SPR and NMR. Int J Biol Macromol..

[22] Tian Y, Grishkewich N, Bromberg L, Hatton TA, Tam KC (2017). Cross-linked Pluronic-g-Polyacrylic acid microgel system for the controlled release of doxorubicin in pharmaceutical formulations. Eur J Pharm Biopharm..

[23] Abdalkader R (2015). Evaluation of the potential of doxorubicin loaded microbubbles as a theranostic modality using a murine tumor model. Acta Biomater..

[24] Gong R, Chen G (2016). Preparation and application of functionalized nano drug carriers. Saudi Pharm J..

[25] Surendra T, Malay KD (2013). Dendrimers and their Applications as Novel Drug Delivery Carriers. J Appl Pharm Sci..

[26] Zhang L (2016). Doxorubicin-loaded polypeptide nanorods based on electrostatic interactions for cancer therapy. J Colloid Interface Sci..

[27] Mezghrani O (2015). Hepatocellular carcinoma dually-targeted nanoparticles for reduction triggered intracellular delivery of doxorubicin. Int J Pharm..

[28] Diaconeasa, Z. *et al*. New insights regarding the selectivity and the uptake potential of nanoceria by human cells. Colloids Surf A Physicochem Eng Asp. 10.1016/j.colsurfa.2017.05.081 (2017).

[29] Zhang P, Kong J (2015). Doxorubicin-tethered fluorescent silica nanoparticles for pH-responsive anticancer drug delivery. Talanta..

[30] Li ZY (2017). Poly (acrylic acid) microspheres loaded with superparamagnetic iron oxide nanoparticles for transcatheter arterial embolization and MRI detectability: *In vitro* and *in vivo* evaluation. Int J Pharm..

[31] Tsai WC (2018). Preparation and characterization of gellan gum/glucosamine/clioquinol film as oral cancer treatment patch. Mater Sci Eng C Mater Biol Appl..

[32] El-Say KM, El-Sawy HS (2017). Polymeric nanoparticles: Promising platform for drug delivery. Int J Pharm..

[33] Rakitina TV (2015). Novel PARP1 inhibitors potentiate doxorubicin antitumor activity *in vitro*. Mendeleev Commun..

[34] Zhou X (2014). *In vitro* and *in vivo* evaluation of chitosan microspheres with different deacetylation degree as potential embolic agent. Carbohydr Polym..

[35] Chen F (2014). Injectable chitosan thermogels for sustained and localized delivery of pingyangmycin in vascular malformations. Int J Pharm..

[36] Fan Y, Yi J, Hua X, Zhang Y, Yang R (2017). Preparation and characterization of gellan gum microspheres containing a cold-adapted β-galactosidase from Rahnella sp. R3. Carbohydr Polym..

[37] Soares PIP (2016). Chitosan-based nanoparticles as drug delivery systems fordoxorubicin: Optimization and modeling. Carbohydr Polym..

